# Neutrophil Count as a Predictor of Critical Coronary Artery Stenosis in Young Patients

**Published:** 2018-05

**Authors:** Ramazan GÜVEN, K. Can AKYOL, Nermin BAYAR, Faruk GÜNGÖR, Ali Haydar AKÇA, Ahmet ÇELIK

**Affiliations:** 1. Dept. of Emergency Medicine, Bitlis State Hospital, Bitlis, Turkey; 2. Dept. of Emergency Medicine, Antalya Training and Research Hospital, Antalya, Turkey; 3. Dept. of Cardiology, Antalya Training and Research Hospital, Antalya, Turkey; 4. Dept. of Emergency Medicine, Van Region Training and Research Hospital, Van, Turkey

## Dear Editor-in-Chief

Inflammation is the pathophysiologic basis of Coronary Artery Disease (CAD) and the first step of this process starts with the thickening of intima, which is the internal layer of the coronary artery wall (1). The elevated endothelial mediators, especially the low-density cholesterol (LDLc) are the earliest inflammatory reaction. These mediators lead to the accumulation of more macrophages and vascular smooth muscle cells and migration of the other inflammatory molecules (platelets and white blood cells). This pathophysiologic process induced by inflammation results in the formation of atherosclerotic plaques (2, 3).

**Fig. 1:**
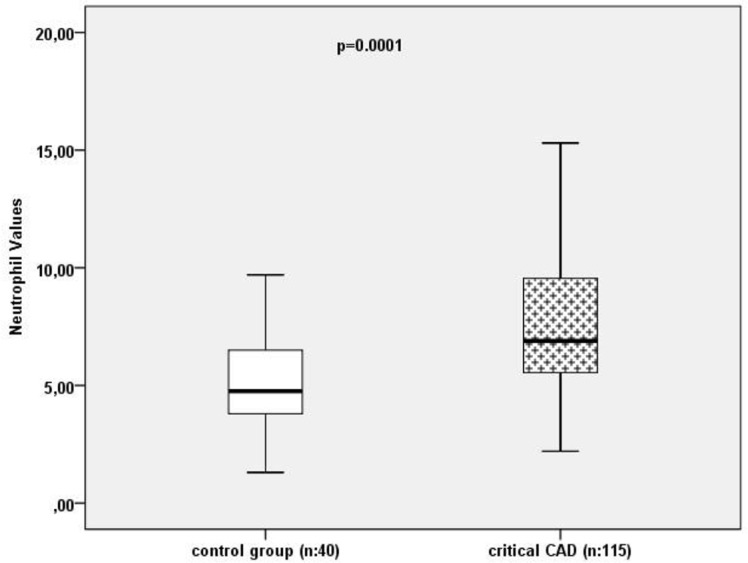
Comparison of neutrophil count among critical CAD patients and control group. (CAD: Coronary artery disease)

Acute Coronary Syndrome (ACS) is a clinical condition that is secondary to the decreased blood flow due to coronary arterial spasm, atherosclerotic plaque rupture and thrombus formation in the area where the atherosclerotic lesion exists (4). There is an increasing body of research showing that lipid and inflammatory molecules could be used to predict ACS and determine the severity of coronary artery obstruction due to their roles in the development of inflammation. In this study, we aimed at exploring the association between the complete blood count subunits and severity of coronary artery obstruction in young patients with ACS using Gensini scoring. Furthermore, we also assessed the diagnostic value of the complete blood count parameters in predicting premature ACS and to what extent it can be used in emergency departments. This retrospective study included patients younger than 45 who presented with chest pain to the Emergency Department of a training and research hospital from 2013 to 2015 and underwent coronary angiography. Informed consent was taken from the participants. Overall, 155 patients were divided into two groups according to the angiography results. The first group included patients who had critical coronary artery stenosis (n=115), while the second group included the controls who did not have critical coronary artery stenosis (n=40). White blood cell (WBC) and neutrophil values were found to be significantly higher in patients with critical CAD compared to the control group (*P*<0.001; 11.61±3.3 vs 8.49±3.0, *P*<0.001; 7.38±3.0 vs 5.43±2.8, respectively) (Fig. 1). WBC, neutrophil and N/L ratio were positively correlated with the Gensini scores (r=0.292; *P*=0.001, r=0.278; *P*=0.002, r=0.209; *P*=0.02, respectively). The multivariate regression analysis revealed that neutrophil count was the only variable associated with critical CAD (OR: 1.297; 95% CI 1.045–1.609; *P*=0.018) (Table 1).

**Table 1:** Effects of various variables on critical coronary artery disease in univariate and multivariate logistic regression analyses

	***Univariate***		***Multivariate***	

**Variable**	**OR (95% CI)**	***P*-value**	**Adjusted OR(95% CI)**	***P*-value**
Age (years)	1.070 (0.999–1.147)	0.054		
Sex	0.196 (0.080–0.483)	<0.001	1.707 (0.358–8.141)	0.502
Neutrophil count	1.375 (1.158–1.634)	<0.001	1.297 (1.045–1.609)	**0.018**
Platelet count	1.008 (1.001–1.014)	0.023	1.002(0.993–1.011)	0.685
LYM	1.397 (0.996–1.960)	0.053		
N/L	1.071 (0.940–1.220)	0.304		
MPV	0.771 (0.577–1.031)	0.079		
Haemoglobin	1.349 (1.088–1.672)	0.006	1.201 (0.869–1.660)	0.268
HT	1.990 (0.745–5.319)	0.170		
HL	4.860 (1.587–14.888)	0.006	0.421(0.106 –1.676)	0.220
Smoking	2.329 (1.056–5.138)	0.036		
DM	5.500 (1.227–24.654)	0.026	6.032 (0.753–48.307)	0.090
Triglyceride	1.006 (1.001–1.010)	0.013	1.003 (0.997–1.008)	0.361
LDL	1.016 (1.006–1.027)	0.003	1.008 (0.995–1.020)	0.239
HDL	0.951 (0.913–9.991)	0.017	0.966(0.910–1.026)	0.264

(WBC: White Blood Cell, N/L: Neutrophil/Lymphocyte, MPV: Mean Platelet Volume, HT: Hypertension, HL: hyperlipidaemia, HDL: high-density lipoprotein, DM: Diabetes Mellitus N/L: Neutrophil/Lymphocyte, HDL: high-density lipoprotein, LDL: Low-Density Lipoprotein)

Complete blood count subunits have been investigated in several studies exploring a wide range of aspects from the severity of CAD to the long-term mortality of CAD since it is cheap and easily accessible. Although majority of the studies focused on N/L ratio (5, 6), we did not find any significant difference in N/L ratio between the critical CAD patients and the control group. However, WBC and neutrophil were markedly higher in critical CAD patients. Furthermore, WBC and neutrophil were found to be significantly associated with the severity of CAD determined using the Gensini score. As a result of the regression analysis in which we used several variables including the classic risk factors of CAD (DM, HT, male, hyperlipidaemia, smoking etc.), neutrophil could be an independent predictor of CAD. Neutrophil could be used for the diagnosis of young patients with critical CAD as an auxiliary diagnostic tool when the neutrophil values were above the cut-off value that we determined for the neutrophil level.

The most important limitation was a single-center study; therefore, it could not be generalized. Furthermore, our study included relatively small sample size and we did not have any data regarding the period prior to CAD.
